# Estrus Synchronization of Replacement Gilts Using Estradiol Cipionate and PGF_2α_ and Its Effects on Reproductive Outcomes

**DOI:** 10.3390/ani12233393

**Published:** 2022-12-02

**Authors:** Diego Feitosa Leal, Carlos Henrique Cabral Viana, Glen William Almond, Matheus Saliba Monteiro, Cesar Augusto Pospissil Garbossa, Rafaella Fernandes Carnevale, Bruno Bracco Donatelli Muro, André Pegoraro Poor, Guilherme Pugliesi, Marcílio Nichi, Tatiane Terumi Negrão Watanabe, Mariana Groke Marques

**Affiliations:** 1 Department of Animal Reproduction, School of Veterinary Medicine and Animal Sciences, University of São Paulo (USP), Pirassununga 13635-900, Brazil; 2 School of Veterinary Medicine, Pontifical Catholic University Minas, Poços de Caldas 37714-620, Brazil; 3 Department of Population Health & Pathobiology, College of Veterinary Medicine, North Carolina State University (NCSU), Raleigh, NC 27607, USA; 4 Department of Preventive Veterinary Medicine and Animal Health, School of Veterinary Medicine and Animal Sciences, University of São Paulo (USP), São Paulo 05508-270, Brazil; 5 Department of Nutrition and Animal Production, School of Veterinary Medicine and Animal Sciences, University of São Paulo (USP), Pirassununga 13635-900, Brazil; 6 Embrapa Suínos e Aves, Concórdia 89715-899, Brazil; 7 Postgraduate Program in Animal Production and Health, Instituto Federal Catarinense, Concórdia 89703-720, Brazil

**Keywords:** estradiol cypionate, prostaglandin, estrous cycle, synchronization, fertility, embryo development, pigs

## Abstract

**Simple Summary:**

The successful introduction of gilts into breeding groups is a key component of overall productivity. Therefore, the development of estrus synchronization protocols that optimize labor and time would benefit pig production. In this study, we evaluated the effectiveness of the estrus synchronization protocol using a single administration of estradiol cypionate on day 12 of the estrus cycle to mimic the maternal recognition of pregnancy in pigs and thus prolong luteal function. Subsequently we treated gilts which displayed prolonged luteal function with prostaglandin F2 alpha (PGF2α) to induce luteolysis and synchronize estrus. We also evaluated the effect of this protocol on fertility and ovarian function. We observed that a single administration of estradiol cypionate at the time of maternal recognition of pregnancy was effective to induce prolonged luteal function, and treatment of these gilts with PGF2α resulted in fertile estrus within a short period of time. Furthermore, no deleterious effects were observed on fertility, follicular development, uterine histoarchitecture, and ovarian function. Based in our results, a single administration of estradiol cypionate followed by PGF2α injection was effective to synchronize estrus in gilts without deleterious effects of reproductive function.

**Abstract:**

In this study, we evaluated the effectiveness of using estrogen-induced prolonged luteal function followed by prostaglandin F2 alpha (PGF2α) treatment to synchronize estrus in gilts. On day12 of the estrus cycle (D0 = first day of standing estrus), 52 gilts were assigned at random to two experimental groups: non-treated gilts (CON, *n* = 22), serving as controls, and prolonged luteal function group (CYP, *n* = 30), receiving a single treatment with 10 mg of estradiol cypionate intramuscularly Starting on day 12, blood samples were collected for estradiol and progesterone assays. Estrus detection started on day 17. Gilts from the CON group were inseminated at the onset of natural estrus. On day 28 CYP gilts were treated with PGF2α to induce luteolysis and inseminated at the onset of estrus. Gilts were slaughtered 5 d after the last insemination. A single treatment with estradiol cypionate prolonged luteal function in 90% of treated gilts. The duration of the estrous cycle was longer (*p* < 0.0001) for CYP gilts compared to CON gilts. CYP gilts showed synchronized estrus 3.96 ± 0.19 d after induction of luteolysis. The conception rate was similar (*p* = 0.10) for CON and CYP gilts. No difference was observed in the embryo recovery rate (*p* = 0.18) and total number of embryos per female (*p* = 0.06). The percentage of unfertilized oocytes, fragmented embryos and viable embryos was similar among females from CON and CYP groups (*p* > 0.05). The treatment of gilts with a single application of 10 mg of estradiol cypionate on day 12 of the estrous cycle was effective in prolonging luteal function and treatment with PGF2α resulted in synchronized estrus. Additionally, the synchronization protocol had no deleterious effect on fertility and embryonic development.

## 1. Introduction

The successful introduction of gilts into breeding groups is an essential component of overall productivity [1,2]. Gilts often stay in the gilt replacement pool for three to four estrous cycles before they are detected in estrus at the right time to fit into a breeding group [3]. The hormonal synchronization of estrus is a management strategy that facilitates the introduction of breeding-eligible females into insemination groups [4]. Therefore, the development of alternative estrus synchronization protocols that optimize labor and time would benefit pig production. In pigs, estrogen stimulation from conceptuses plays a fundamental role in the establishment of pregnancy [5,6,7]. Two phases of estrogen stimulation from conceptuses are required for pregnancy to be established [8]. Pig embryos secrete estrogens between 11–12 days of gestation providing the initial signal for the maternal recognition of pregnancy [9]. The second phase of embryonic estrogen production occurs between 15–30 days, which is required for the maintenance of pregnancy [6]. The exogenous administration of estrogens at the time of maternal recognition of pregnancy is a means to extend luteal function [10]. After PGF2α treatment, gilts with prolonged luteal function will exhibit estrus within a given period [3,10,11,12,13,14]. Indeed, the repeated administration of estradiol benzoate (EB) [13], estradiol valerate (EV) [15], or single administration of estradiol dipropionate (EDP; Ovahormone Depot, Aska pharmaceutical, Tokyo, Japan; 20 mg i.m) [12,16,17] at the time of pregnancy recognition extended luteal function in female pigs, with females exhibiting estrus 3–7 days after PGF2α treatment. However, the need for multiple administrations of EB or EV makes the protocol impractical, and EDP is a human pharmaceutical [12].

Estradiol cypionate (EC) is an estradiol ester with low solubility in water and slow release from the administration site, which prolongs its plasma concentrations for more than 10 days [18]. A convenient protocol to synchronize estrus of replacement gilts could be obtained by a single treatment of cyclic gilts with EC at the time of maternal recognition of pregnancy, extending luteal function. After the induction of luteolysis with PGF2α, estrus would be synchronized. To the best of our knowledge, no studies have been conducted in which there was an evaluation of EC in association with PGF2α to synchronize estrus in replacement gilts. The present study tested the hypothesis that prolonged luteal function can be realized by treating cyclic gilts with EC in a single dose on day 12 of the estrous cycle, and the treatment of gilts that display prolonged luteal function with PGF2α will synchronize estrus. Further, we evaluate the effects of the estrus synchronization protocol on fertility, embryo and follicular development, and on endometrial glandular epithelium.

## 2. Materials and Methods

### 2.1. Locality, Animals, Housing, and Feeding

The study was carried out in the experimental swine complex at Embrapa Suínos e Aves located at Santa Catarina State, Brazil (27°18’48”7”S, 51°59’34”07”W. Fifty-two gilts were used: 25 females from the lineage MS115, Embrapa (Piétrain x Large white x Duroc) and 27 crossbred females (Large white x Landrace) with an average age of 220 ± 5 days, 140 ± 3 kg of live weight, and at least three estrous cycles. During the entire experimental period, gilts were housed in collective pens (four gilts/pen) with partially slatted concrete floors and nipple drinkers. Gilts were fed a standard corn soybean diet (3315 kcal ME/kg, 15.7% crude protein, and 0.9% digestible lysine) twice a day, totaling 2.4 kg/d. Ad libitum access to water was provided throughout the experimental period. 

### 2.2. Experimental Design 

**Figure 1:**
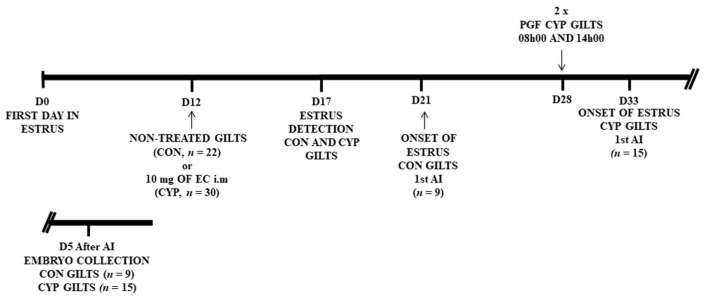
Schematic representation of the experimental design; On day12 of the estrus cycle (D0 = first day of estrus), 52 gilts were assigned at random to two experimental groups: non-treated gilts (CON, *n* = 22) and treatment with 10 mg of estradiol cypionate (CYP, *n* = 30), a single treatment with 10 mg of estradiol cypionate i.m. Starting on day 17, Gilts from both experimental groups were check for signs of estrus. CON gilts (*n* = 9) were artificially inseminated (AI) at onset of estrus around day 21, and euthanized for embryo collection five days (D5) after insemination. CYP gilts were treated with sodium cloprostenol (PGF) twice (first dose: 08h00; second dose: 14h00). CYP gilts (*n* = 15) were artificially inseminated (AI) at onset of estrus around day 32 (D0 first day of previous estrus), and euthanized for embryo collection five days (D5) after insemination.

On day 12 of the estrous cycle (D0 = first day of estrus), 52 gilts were assigned at random into two experimental groups: Control (*n* = 22) without application, and CYP (*n* = 30) treatment with 10 mg of estradiol cypionate (SincroCP^®^ 10 mg/mL of estradiol cypionate, Ourofino Saúde Animal, Cravinhos SP, Brazil) intramuscularly (Figure 1). Blood samples were collected to determine the hormonal concentrations of progesterone and estradiol starting on day 12 (before application of EC) and every 72 h. On day 28 (D0 = first day in estrus), gilts with prolonged luteal function were treated twice with sodium cloprostenol (first dose: 0.263 mg at 08h00; second dose: 0.263 mg of at 14h00; Sincrocio^®^, Ourofino Saúde animal, Cravinhos SP, Brazil) intramuscularly for the induction of luteolysis. The prolonged luteal function was defined as the absence of signs of estrus and maintenance of progesterone (P4) level above 1 ng/mL between day 12 and 28. To evaluate the effects of the protocol on fertility, a subgroup composed of 24 MS115 gilts (CON, *n* = 9 and CYP, *n* = 15) were inseminated and slaughtered five days after the last insemination. The remainder of the gilts (Large white x Landrace) were not slaughtered. These gilts were inseminated and used by the farm in which this experiment was conducted.

### 2.3. Ultrasound Scanning 

An ultrasound scanning of the ovaries was performed by a single trained technician (D.F.L.) using real-time transabdominal ultrasonography with a 5 MHz sectorial transducer (Imago.S, IMV Immaging UK Ltd., Bellshill, UK). The ultrasound evaluation was carried out at two moments: on the first day of the experiment (day 12 of the estrous cycle) to confirm that the females from the CON and CYP groups were at similar stages of the estrous cycle and again on the first day of observed standing estrus to measure the diameter of the follicles at onset of estrus. The mean of the three largest follicles only from the right ovary was used to determine follicle diameter, as described elsewhere [19,20].

### 2.4. Estrus Detection and Artificial Insemination

Estrus detection started on day 17 of the estrous cycle for both experimental groups. All gilts were checked for signs of estrus twice daily (08h00 am 16h00) by direct contact with a mature boar and a back-pressure test. Gilts from the CON group (*n* = 9) were inseminated at the onset of natural estrous. CYP gilts (*n* = 15) were inseminated at the onset of estrus following the induction of luteolysis. All gilts were inseminated (intracervical insemination) at first signs of standing estrus and every 24 h with fresh diluted semen (3 × 10^9^ sperm cells; > 80% motility) kept at 17 °C. The semen of the same boar was used within each batch, thus the females from the CON and CYP groups were inseminated with the same ejaculate. The inseminations were conducted in the presence of a sexually mature boar. Gilts received in average of 2.4 ± 0.6 inseminations.

### 2.5. Progesterone and Estradiol Assays

Blood samples were obtained on day 12 of the estrous cycle and every 72 h until day 29 for CYP gilts or until estrus was observed for the first time for the CON gilts. For P4 and 17β-estradiol (E2) assays, blood samples were collected by jugular puncture using polypropylene tubes without an anticoagulant. The samples were centrifuged at 1300× *g* for 15 min at 4 °C, and the serum obtained was aliquot in duplicate and stored at −20 °C until assay. The serum progesterone and 17β-estradiol levels were determined by the solid phase radioimmunoassays using commercial kits (Beckman Coulter, Brea, USA). The hormonal assays were performed according to the protocol provided by the manufacturer. The progesterone measurements were analyzed in duplicate. The intraassay CV was 7.35%, and the interassay CV was 7.25%. The average sensitivity for the P4 assay was 0.5 ng/mL. The samples for E2 were analyzed in a simple assay. The intraassay and interassay CVs were 6.01% to 7.00%. The average sensitivity for the E2 assay was 0.06 pg/mL. Serum concentration of E2 was only determined for the CYP group.

### 2.6. Embryo Recovery, Conception, and Ovulation Rate

A subgroup composed of 24 gilts (MS115, Pietrain x Large white x Duroc) were slaughtered five days after insemination (D0 = last artificial insemination) at a local abattoir. The embryos were collected by uterine flushing [21]. Embryo quality and developmental stage were assessed according to the criteria established by the International Embryo Transfer Society (IETS) as described by [22]. The conception rate was determined by the presence of embryos in uterine horn flushing. The ovulation rate was assessed by counting the number of corpora lutea on both ovaries.

### 2.7. Histology of Uterine Glandular Epithelium

On day five of pregnancy, uterine fragments measuring approximately 4 cm^2^ were collected from the central region of the left uterine horn; the central region was chosen to standardize the analysis. Glandular epithelium evaluations were performed as described by [23]. After collection, the samples were stored in 10% buffered formalin for 24 h and then processed for histology. For this purpose, sections (4 μm) were obtained and stained with hematoxylin and eosin. Three images per slide were saved using the 4x objective (total area of each image = 7.45 mm^2^). The images were captured as “jpeg” files using a microscope (Leica DM500) equipped with a high-definition camera (Leica ICCD50 HD). Glandular density (GD, glands/mm^2^) was determined by the number of glands divided by the endometrial area in the observed fields. The endometrial area was measured individually for each histological field, from the top of the luminal epithelium to the myometrium, using the freehand selections tool of the ImageJ^®^ software (version 1.51v). The mean glandular area (MGA, µm^2^) was defined as the mean size of each glandular structure. The average area of 50 glands per photomicrograph was measured using ImageJ^®^ software. 

### 2.8. Statistical Analysis

The statistical analyses were performed in SAS software version 9.4 (SAS/STAT, SAS Institute Inc., Cary, NC, USA). All residues were tested for normality and homogeneity. The PROC RANK procedure with the NORMAL option produced a transformed variable normalized for variables that did not follow a normal distribution. Plasma concentrations of P4 and E2 over time were analyzed with repeated measures using the SAS PROC MIXED procedure. All data were described as LSMEANS and the largest standard error (SEM) of each variable was used. The means were compared with a Tukey test. Differences were considered significant if *p* < 0.05. The number of corpora lutea and data from endometrial glands evaluation were compared with a t test. The recovery rate was analyzed with generalized mixed models where the number corpora lutea was included as a random effect. The variables related to embryonic development and conception rate were analyzed with generalized mixed models where the embryo development stage was the dependent variable and the number of corpora lutea was included in the model as a random effect. The variation of the estrous cycle duration was analyzed with generalized mixed models. All models considered the treatment group as a fixed effect, and when different genetic lines were used, it was included as a random effect. Differences were considered significant when *p* < 0.05.

## 3. Results

### 3.1. Induction of Prolonged Luteal Function, Estrus Synchronization, and Hormone Profile

**Figure 2:**
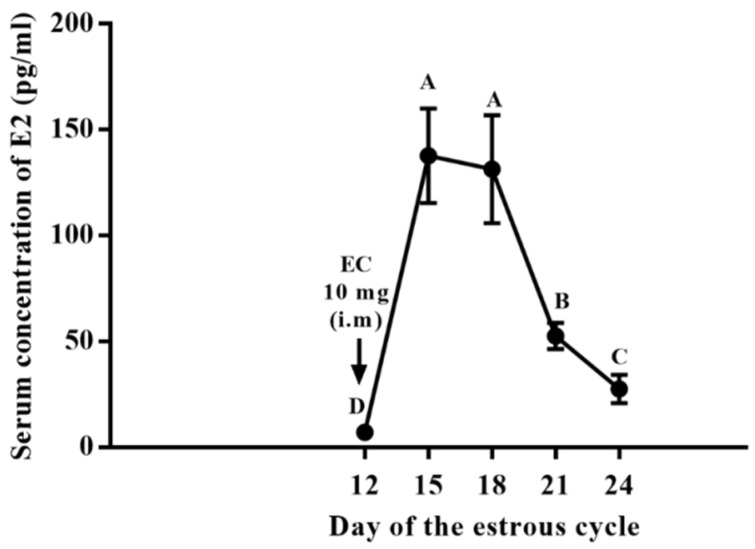
Serum concentration of E2 after estradiol cypionate administration. Capital letters indicates statistical difference (*p* < 0.05).

**Figure 3:**
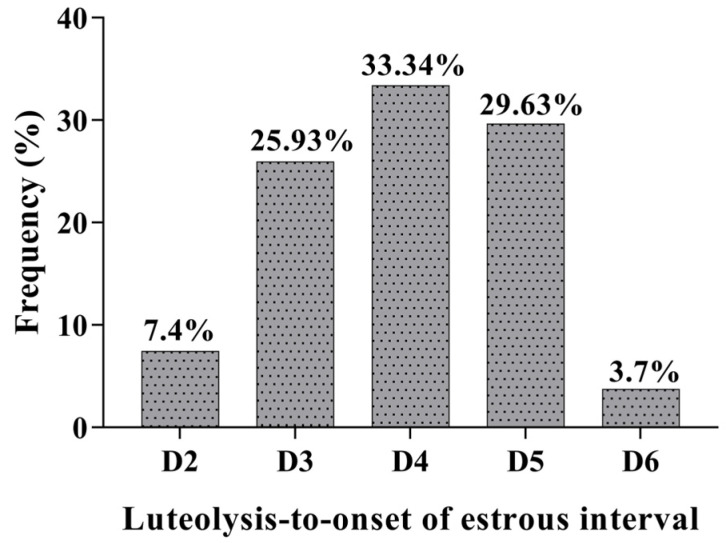
Intervals from sodium cloprostenol treatment to onset of estrus in gilts with prolonged luteal function (CYP gilts). The data are presented as frequency per total number of gilts.

**Figure 4:**
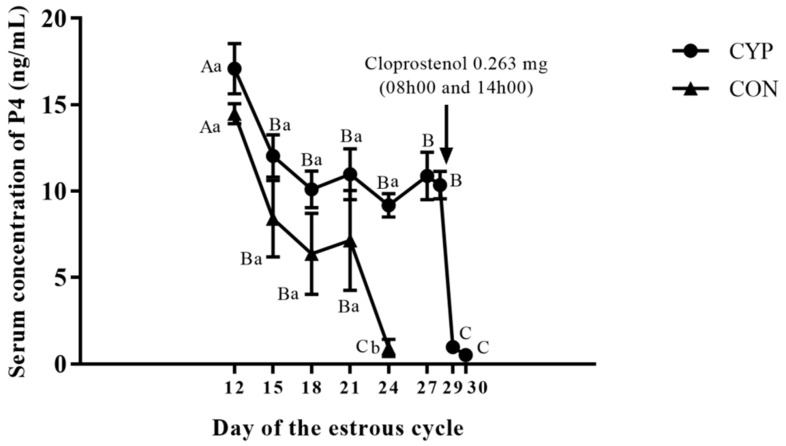
Serum concentration of P4 over the estrous cycle in the CYP and CON groups. CYP: Cypionate group; CON: control gilts. Capital letters indicate statistical difference (*p* < 0.05) over time whiting experimental group; small letters indicate statistical difference (*p* < 0.05) between experimental groups in the correspondent day of the estrous cycle.

The treatment with a single application of estradiol cypionate on day 12 of the estrous cycle induction caused a prolonged luteal function in 90% (27/30) of gilts in the CYP group. The Inter-estrous interval was longer (*p* < 0.001) for gilts in the CYP group compared to gilts in the CON group (Table 1). Two gilts treated with EC on day 12 of the estrus cycle were already in estrus on the day of the induction of luteolysis with sodium cloprostenol. The serum concentration of E2 of gilts in the CYP group remained high for six days after treatment with EC (Figure 2). All gilts with prolonged luteal function showed synchronized estrus on average 3.96 ± 0.19 days after the induction of luteolysis (Figure 3). The P4 concentration of gilts in the CYP group remained above 5 ng/mL from days 12 to 28 (*p* < 0.001); conversely, for gilts in the CON group, the P4 concentration declined to values below 1 ng/mL on day 21 of the estrous cycle (*p* = 0.003). The P4 concentration of CYP gilts declined (*p* < 0.001) to values below 1 ng/mL 24 h after the induction of luteolysis (Figure 4). The duration of estrus did not differ between experimental groups (CON, 2.32 ± 0.16; CYP, 2.24 ± 0.16; *p* = 0.16).

### 3.2. Effect of the Synchronization Protocol on Fertility, Embryo, and Follicular Development

The embryo recovery and development outcomes are summarized in Table 2. The conception rate did not differ between experimental groups (CON, 88.9%; CYP 93.3%; *p* = 0.94). The number of ovulations was similar between the CON and CYP groups (*p* = 0.17). No difference was observed for the embryo recovery rate (*p* = 0.47). For the analysis of embryonic development, the percentage of unfertilized oocytes, fragmented embryos, and viable embryos were similar among CON and CYP gilts. The mean diameter of the follicles on day 12 of the estrous cycle was similar between the experimental groups (*p* = 0.58). Conversely, the mean diameter of follicles at the beginning of subsequent estrus was greater (*p* < 0.001) for CYP gilts compared to CON gilts (Table 3).

### 3.3. Effect of the Estrus Synchronization Protocol on the Uterine Glandular Epithelium

**Figure 5:**
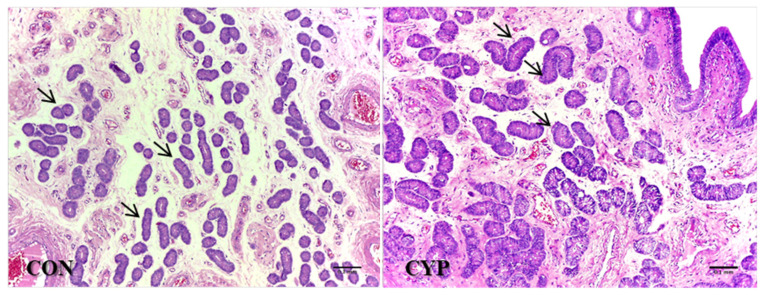
Histology of uterine glandular epithelium of gilts slaughtered on day 5 after insemination; gilts were inseminated at natural estrus (CON) or following induced delayed luteal regression (CYP). The mean glandular area (MGA) was higher (*p* = 0.04) for the CYP group compared to the CON group (arrows).

The mean area of endometrial glands was higher (*p* = 0.04) for the CYP group compared to the CON group (Figure 5). No differences were observed between the experimental groups for the following variables: glandular density (CON, 75.517 ± 4.2; CYP, 84.798 ± 3.061; *p* = 0.08) and endometrial area (CON, 1811.13 ± 100.750; CYP, 1790.36 ± 102.760; *p* = 0.89).

## 4. Discussion

The present study demonstrated the effectiveness of the estrus synchronization protocol using a single administration of EC on d 12 of the estrus cycle combined with treatment with cloprostenol to synchronize estrus in replacement gilts. Similarly, [12] demonstrated that a single administration of 20 mg of EDP between 8–11 days after ovulation resulted in 90% of the gilts having prolonged luteal function, with 100% of these gilts showing synchronized estrus between 5.5 ± 0.1 days after PGF2α treatment. Repeated treatment with 5 mg of EV from days 11–15 (D0 = first day in estrus) resulted in 100% of gilts presenting prolonged luteal function, with 92% of these females in standing estrus 4.9 ± 0.2 days after treatment with PGF2α [15]. Noguchi et al. [11] observed that a single treatment of 20 mg EDP on day 12 of the estrous cycle (D0 = first day of estrus) prolonged luteal function in 82% of the treated gilts, with 83% of the females in estrus 5.7 ± 0.3 days after treatment with PGF2α. Altogether, these results strengthen the notion that estrus can be synchronized in gilts by associating estrogen-induced delayed luteal regression and the induction of luteolysis with PGF2α. Furthermore, the success of the protocol is closely related to the moment of the estrous cycle in which gilts are treated with estrogen. In pregnant pigs, E2 concentrations in the uterine lumen increase biphasically (11–12 and 15–30 days of gestation), representing the first and second signals for the maternal recognition of pregnancy [24]. Research efforts have shown that increased concentrations of estrogen during these periods are necessary to delay luteal regression in pigs [13,15,17]. Thus, administration of estradiol outside these periods would compromise the rate of delayed luteal regression induction and the number of synchronized gilts. Indeed, the treatment with E2 before day 9 and after day 16 of the estrous cycle (D0 = first day in estrus) did not extend corpus luteum activity [10]. Conversely, luteal regression was delayed for 60 day when gilts were treated with 10 mg of EB from day 11 to 15 of the estrus cycle [13]. However, a single treatment of EB 9.5, 11, or 12.5 days after the onset of estrus did not prolong luteal function [10]. In our study, gilts were treated with EC on day 12 of the estrous cycle, and the first day in estrus was considered as the D0, which would correspond approximately to 10–11 days after ovulation [25]. Concurrently, we observed that serum E2 concentrations remained high during the period required for the establishment of gestation in pigs. It is worth mentioning that we chose not to use ultrasound scanning to monitor ovulation and thus use the time of ovulation as D0, so that the protocol could be more applicable in commercial pig operations. Considering the first day in estrus of replacement gilts as D0 minimizes the chances of administering EC outside the optimal period required to prolong luteal function, regardless of estrus length and, consequently, the time of ovulation. 

The P4 concentration of gilts treated with EC remained above 5 ng/mL from day 12 to 28, declining from 9.63 ng/mL to less than 1 ng/mL one day after induction of luteolysis. Our results were similar to those observed in gilts treated with multiple applications of EB [13] or a single application of EDP [11,12] to delay luteal regression. In these studies, the decline of P4 to less than 1ng/mL occurred 21–57 h and 24–48 h after treatment with PGF2α, respectively. The P4 concentration should decline sharply after luteolysis. The maintenance of relatively high P4 concentrations after the regression of corpus luteum interferes with LH pulsatility. Under this circumstance, LH release is strong enough to induce follicle development until the antral stage, however insufficient to induce ovulation, and these follicles tend to grow into cysts [26,27].

In our study, no differences in the duration of estrus were observed for CON and CYP gilts. Moreover, the duration of estrus was within the normal range described for gilts [28].This result further strengthens the absence of deleterious effects of the protocol on ovarian activity due to endocrine abnormalities, which is among the factors that can affect the duration of estrus [26]. Our results were similar to those found in other studies using estradiol and PGF2α to synchronize estrus. Indeed, Noguchi et al. [11] reported that the duration of estrus for females in the treatment and control groups was 2.3 ± 0.3 and 2.3 ± 0.2 days, respectively.

The diameter of the follicles on day 12 of the estrous cycle was similar between gilts in both experimental groups. Conversely, at the beginning of estrus, CYP gilts had larger follicles compared to CON gilts. The increase in follicles diameter at the preovulatory phase is associated with improvement in intrafollicular microenvironment, with repercussions on oocyte quality and blastocyst uniformity [29,30,31]. Evidence from the studies demonstrated that the quality of ovulated oocytes plays a key role in embryonic development [30,32]. Xie et al. [33] demonstrated that oocytes with a lower degree of maturation resulted in lesser developed zygotes, which are the first embryos eliminated when the uterine capacity becomes limiting for conceptus development. Nonetheless, in our experiment, the increase in the size of follicles during the preovulatory phase had no effect on early embryonic development.

The protocol for estrus synchronization used in our experiment had no deleterious effect on fertility as no difference in conception rate between the experimental groups was observed. Additionally, the conception rate of CYP gilts is in accordance with values considered ideal [34]. The evaluation of embryonic development showed that the percentage of viable embryos collected on day 5 after insemination was similar between experimental groups. Similarly, Hirayama et al. [16] observed that the viable embryo rate was 80% in gilts synchronized using EDP and PGF2α. 

In gilts, another benefit associated with the use of estrus synchronization protocols based on the induction of prolonged luteal function is an improvement in litter size and weight [13,15]. The increased litter quality might be due to enhanced endometrial function resulting from prolonged exposure of the endometrium to P4 [15]. In the present experiment, CYP gilts had increased mean glandular area compared to CON gilts. Studies have already shown that the increase in the area of uterine glands was accompanied by increased expression of IGF-I, VEGF, and increased weight of embryos collected on day 28 of gestation [23,35]. Nevertheless, in our study, the increase in the mean glandular area was not accompanied by changes in embryonic development, possibly because of the stage of embryo development in which the embryos were collected. Further studies are warranted to evaluate whether the increase in mean glandular area could have any beneficial effects on embryos at more advanced stages of development.

The major limitations of our study are that the use of estradiol cypionate is not approved for use in pigs in several countries. Therefore, while the estrus synchronization protocol using estradiol cypionate in combination of PGF2α effectively synchronized estrus in replacement gilts without deleterious effects on reproductive function, its application in commercial pig operations is still limited due to current legislation. For countries that do not permit the use of estradiol cypionate in pigs, the protocol tested here could be used in research settings, legislation permitting. 

## 5. Conclusions

The estrus synchronization protocol used in the present experiment yielded encouraging results. Estrus in gilts was synchronized through a single application of EC and two applications of PGF2α with more than 90% effectiveness. Furthermore, no deleterious effects were observed for ovarian function or fertility. The protocol used in the present experiment could be another alternative for estrus synchronization in replacement gilts. Since we observed that gilts submitted to the estrus synchronization protocol had increased follicle size at the onset of estrus and higher mean glandular area, further studies are warranted to evaluate the effects of the protocol on embryo/fetal development and litter traits. 

## Figures and Tables

**Table 1:** Duration of the estrous cycle and estrus of gilts subjected to the estrus synchronization protocol using estradiol cypionate and PGF2α.

Variables	Treatments		SEM	*p*
	CON (*n* = 21)	CYP (*n* = 30)		
Inter-estrus interval ^1^ (days)	20.67 ^a^	31.69 ^b^	0.320	<0.0001
Estrus duration (days)	2.32	2.24	0.161	0.72

CON, Control group; CYP, Cypionate group (gilts treated with 10 mg of estradiol cypionate on day 12 of estrus cycle and 0.526 mg of sodium cloprostenol on day 28). ^1^ Inter-estrus interval is the interval between the previous and the subsequent estrus. Means within rows with different superscript differ (*p* < 0.05).

**Table 2:** Fertilization rate (%), embryo recovery rate (%), unfertilized oocytes (%), fragmented embryos (FE; %), 4–8 cells embryos (%), 8–16 cells embryos (%), morula (M; %), compact morula (CM; %), early blastocyst (EB; %), expanded blastocyst (XB; %) and hatched blastocyst (HB; %) of gilts subjected to estrus synchronization protocol using estradiol cypionate and PGF2α.

	Variables					Embryo Development Stage						
	Conception Rate	Recovery Rate	Unfertilized Oocytes		FE	4–8 Cells	8–16 Cells	M	CM	EB	XB	HB
CON (*n* = 9)	8 (88.8)	78 (77.0)	14 (17.9)		1 (1.5)	14 (21.8)	5 (7.8) ^a^	20 (31.2) ^a^	8 (12.5)	5 (7.8)	7 (10.9)	4 (6.2)
CYP (*n* = 15)	14 (93.3)	163 (87.5)	19 (11.6)		7 (4.8)	34 (23.6)	33 (22.9) ^b^	24 (16.6) ^b^	29 (20.1)	5 (3.4)	3 (2.1)	9 (6.2)

CON, Control group; CYP, Cypionate group (gilts treated with 10mg of estradiol cypionate on day 12 of estrus cycle and.0.526 mg of sodium cloprostenol on day 28. Means within rows with different superscript differ (*p* < 0.05).

**Table 3:** Number of ovulations and mean follicle size on day 12 of the estrous cylce (D12) and at estrus onset (EO) of gilts subjected to estrus synchronization protocol using estradiol cypionate and PGF2α (Mean ± SEM).

Variables	CON (*n* = 9)	CYP (*n* = 15)	*p*
Number of ovulations (n)	12.5 ± 0.5	14.0 ± 0.90	0.17
Mean follicle size at D12 * (mm)	2.2 ± 0.5	2.3 ± 0.3	0.58
Mean follicle size at EO * (mm)	7.47 ± 0.40 ^a^	8.16 ± 0.60 ^b^	<0.001

CON, Control group; CYP, Cypionate group (gilts treated with 10mg of estradiol cypionate on day 12 of estrus cycle and 0.526 mg of sodium cloprostenol on day 28. * Average of the largest three follicles from the right ovary. Means within rows with different superscript differ (*p* < 0.05)

## Data Availability

The data sets and materials are available from the corresponding author upon reasonable request.
